# Halotolerant bacteria mitigate the effects of salinity stress on soybean growth by regulating secondary metabolites and molecular responses

**DOI:** 10.1186/s12870-021-02937-3

**Published:** 2021-04-12

**Authors:** Muhammad Aaqil Khan, Atlaw Anbelu Sahile, Rahmatullah Jan, Sajjad Asaf, Muhammad Hamayun, Muhammad Imran, Arjun Adhikari, Sang-Mo Kang, Kyung-Min Kim, In-Jung Lee

**Affiliations:** 1 School of Applied Biosciences, Kyungpook National University, Daegu, 41566 Republic of Korea; 2 Natural and Medical Plants Research center, University of Nizwa, 616 Nizwa, Oman; 3 Department of Botany, Abdul Wali Khan University, Mardan, Pakistan

**Keywords:** Halotolerant PGPR, Salinity stress, Phytohormones, Antioxidants, Gene expression, Soybean

## Abstract

**Background:**

Salinity is a major threat to the agriculture industry due to the negative impact of salinity stress on crop productivity. In the present study, we isolated rhizobacteria and evaluated their capacities to promote crop growth under salt stress conditions.

**Results:**

We isolated rhizospheric bacteria from sand dune flora of Pohang beach, Korea, and screened them for plant growth-promoting (PGP) traits. Among 55 bacterial isolates, 14 produced indole-3-acetic acid (IAA), 10 produced siderophores, and 12 produced extracellular polymeric and phosphate solubilization. Based on these PGP traits, we selected 11 isolates to assess for salinity tolerance. Among them, ALT29 and ALT43 showed the highest tolerance to salinity stress. Next, we tested the culture filtrate of isolates ALT29 and ALT43 for IAA and organic acids to confirm the presence of these PGP products. To investigate the effects of ALT29 and ALT43 on salt tolerance in soybean, we grew seedlings in 0 mM, 80 mM, 160 mM, and 240 mM NaCl treatments, inoculating half with the bacterial isolates. Inoculation with ALT29 and ALT43 significantly increased shoot length (13%), root length (21%), shoot fresh and dry weight (44 and 35%), root fresh and dry weight (9%), chlorophyll content (16–24%), *Chl a* (8–43%), *Chl b* (13–46%), and carotenoid (14–39%) content of soybean grown under salt stress. Inoculation with ALT29 and ALT43 also significantly decreased endogenous ABA levels (0.77-fold) and increased endogenous SA contents (6–16%), increased total protein (10–20%) and glutathione contents, and reduced lipid peroxidation (0.8–5-fold), superoxide anion (21–68%), peroxidase (12.14–17.97%), and polyphenol oxidase (11.76–27.06%) contents in soybean under salinity stress. In addition, soybean treated with ALT29 and ALT43 exhibited higher K^+^ uptake (9.34–67.03%) and reduced Na^+^ content (2–4.5-fold). Genes involved in salt tolerance, *GmFLD19* and *GmNARK*, were upregulated under NaCl stress; however, significant decreases in *GmFLD19* (3–12-fold) and *GmNARK* (1.8–3.7-fold) expression were observed in bacterial inoculated plants.

**Conclusion:**

In conclusion, bacterial isolates ALT29 and ALT43 can mitigate salinity stress and increase plant growth, providing an eco-friendly approach for addressing saline conditions in agricultural production systems.

**Supplementary Information:**

The online version contains supplementary material available at 10.1186/s12870-021-02937-3.

## Background

Soil salinity is among the most devastating environmental stressors that negatively affect crop production, affecting millions of hectares of land around the world and causing significant economic losses each year [1–3]. Approximately 62 million hectares (20%) of irrigated land are currently affected by high salt content [1, 2], and it has been estimated that more than 50% of arable land will be salinized by 2050 [4, 5]. Soil salinity limits crop productivity by impairing root growth, nutrient uptake, and metabolic processes [1–3]. The active rhizosphere zone is reduced as a result of impaired root growth and development, affecting nutrient uptake efficiency.

In addition, salinity stress affects physiological, morphological, and biochemical processes, which decrease crop biomass and productivity [2, 3]. Excess Na and Cl ions result in ionic imbalances and ion toxicity through competition with K ions. Ion toxicity interferes with many physiological processes in plants and leads to chlorosis and necrosis [6, 7]. Similarly, Na inhibits important cellular processes and enzymatic activities that require K for functioning by competing with K for binding sites and disturbing K homeostasis [6]. It has been suggested that plant survival under salinity stress requires high cytosolic K concentrations and maintenance of low Na concentrations in the cytoplasm and cytosol [8, 9]. Morphological changes under salinity stress have been observed in all growth stages, including germination, seedling, vegetative, and mature stages [10]. Salt stress-induced biochemical changes include the modulation of phytohormones (decreases in the stress hormone abscisic acid [ABA] and increases in the defense hormone salicylic acid [SA]), changes in ion uptake (accumulation or removal of ions), antioxidant enzyme activation, reactive oxygen species (ROS) generation and accumulation, and photosynthetic pathway disruption [2]. On a molecular level, salinity stress in plants also affects gene regulation. Furthermore, salinity stress impedes photosynthesis by affecting chlorophyll and carotenoids and reducing PSII activity [11–13]. A decline in photosynthesis will eventually deplete energy reserves, leading to plant starvation, foliar expansion of leaves, and senescence [14].

Soybean is an economically important legume crop that is cultivated for plant oil, mineral, and protein resources worldwide [5, 15]. Soybean is semi-tolerant to salinity stress; however, high salinity can decrease soybean yield by inhibiting seed germination and post-germination growth [16–18]. Salinity stress negatively impacts soybean growth, seed quality and quantity, and yield [19]. For soybean, the availability of the whole genome sequence has enhanced our understanding of the basic mechanisms of salinity-related gene expression and regulation [20]. Several soybean genes that confer salinity stress tolerance have been identified [21]. *GmFLD19* has been shown to enhance tolerance to salt stress by reducing Na ion and malondialdehyde content, upregulating antioxidant enzyme activity, and increasing chlorophyll content [15]. Similarly, *GmNARK* induces ABA production, improving tolerance to salt stress in soybean [22].

Saline conditions present major challenges for agricultural production in many countries [23–25]. Strategies such as traditional breeding, genetic engineering of halotolerant transgenic plants, and chemical applications are used to address the issue of high salinity [26, 27]. However, such methods are not always feasible, and some may even create additional adverse impacts on the ecosystem [28]. Therefore, identifying and developing ecofriendly strategies to manage high salinity are vital for agricultural systems. The use of plant growth-promoting rhizobacteria (PGPR) to elicit mechanisms facilitating plant tolerance to salinity stress has emerged as a promising approach to improve plant adaptation and resource-use efficiency in hostile environments [17, 18, 29, 30]. PGPR can benefit plant growth and development by producing phytohormones and organic substances that promote growth and increase nutrient availability and uptake [17, 23]. Several studies have reported the effectiveness of PGPR for improving crop growth under abiotic stress, including salinity stress. Bacterial strains such as *Pseudomonas*, *Burkholdera, Bacillus*, and *Arthrobacter* have been identified as plant growth promoters under saline conditions in crop plants such as cucumber, tomato, wheat, and soybean [23, 29–31]. The purpose of the current study was to isolate, identify, and characterize halotolerant PGPRs. Bacterial isolates ALT29 and ALT43 were selected based on multiple plant growth promotion (PGP) traits. We evaluated the effects of halotolerant isolates ALT29 and ALT43 on soybean growth attributes, ion uptake, ROS generation and antioxidant production, and salt-related gene expression under NaCl stress at concentrations of 80 mM, 160 mM, and 240 mM.

## Results

### Isolation, screening, and identification

**Fig. 1:**
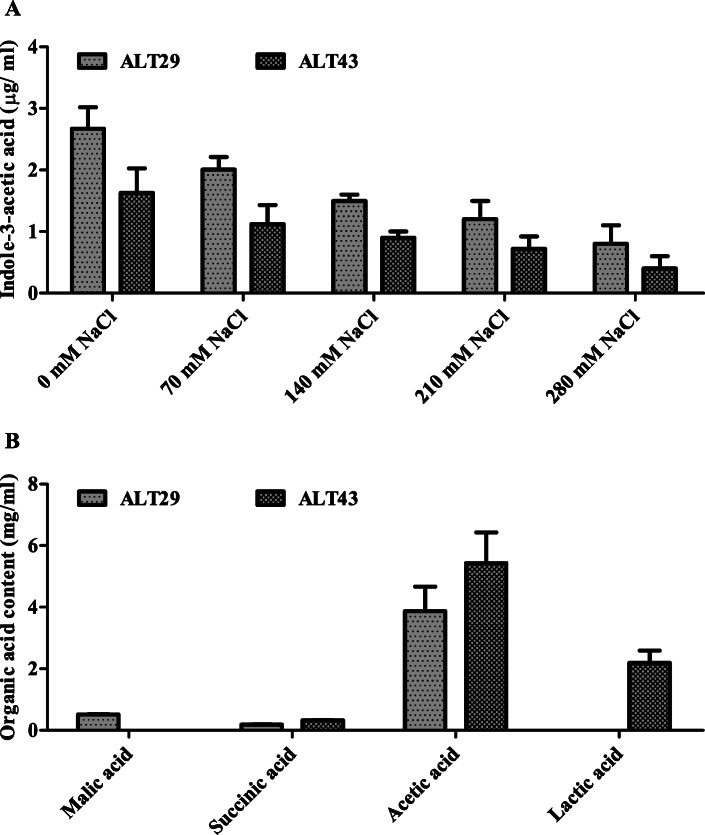
Quantification of **a** IAA and **b** organic acid content in isolates ALT29 and ALT 43 culture broth using GC/MS-SIM analysis. Data points are the mean of three technical replications and error bars represent standard error. Bars with different letters are significantly different from each other, as evaluated by DMRT analysis

**Fig. 2:**
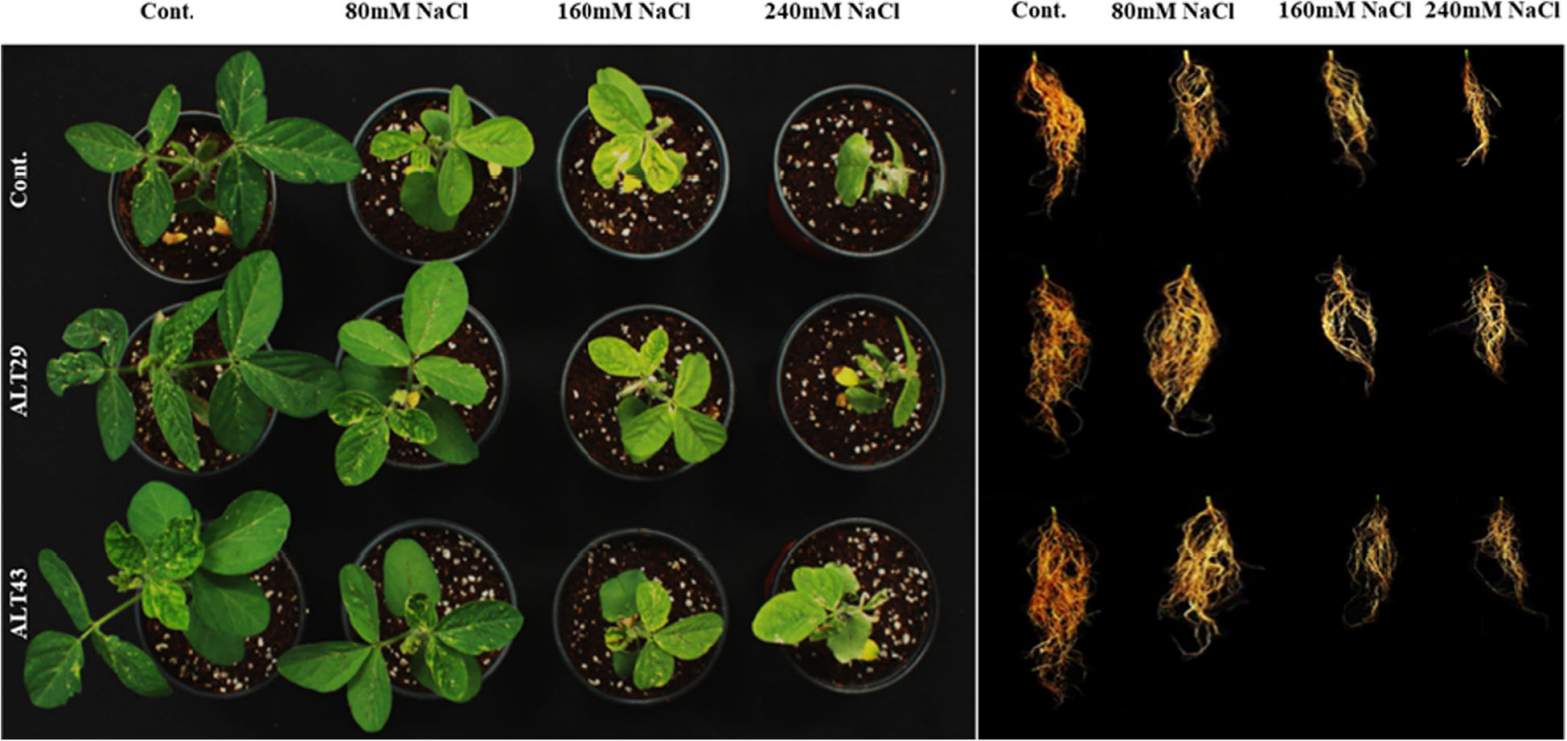
Effects of bacterial isolates ALT29 and ALT 43 on the growth of soybean plants under normal conditions and NaCl stress

**Fig. 3:**
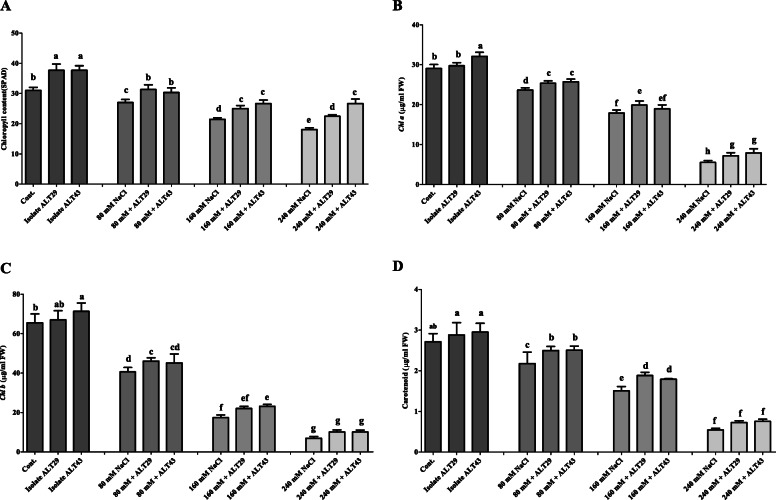
Effects of NaCl stress on chlorophyll content in soybean plants with and without inoculation of isolates ALT29 and ALT 43. **a** Chlorophyll content (SPAD), **b** Chlorophyll A (Chl A), **c** Chlorophyll B (Chl B), and **d** Carotenoid content. Data points are the mean of three technical replications and error bars represent standard error. Bars with different letters are significantly different from each other, as evaluated by DMRT analysis

Fifty-five bacterial isolates were isolated (S. Table 1) and screened for plant growth-promoting traits (indole-3-acetic acid [IAA], siderophore production, extracellular polymeric substances [EPS] formation, phosphate solubilization, and NaCl tolerance). The Salkowski reagent results revealed that 14 isolates exhibited IAA activity, 12 isolates exhibited EPS activity and phosphate solubilization, and 10 isolates produced siderophores (S. Fig. 1). Based on multiple PGP traits, we selected 11 isolates to screen at different concentrations of NaCl (70 mM, 140 mM, 210 mM, and 280 mM). The highest NaCl tolerance was observed in ALT29 and ALT43 (S. Fig. 2), which were, therefore, selected for further investigation and molecular identification. The identification results showed that ALT29 and ALT43 exhibit high sequence similarity with *Bacillus aryabhattai* and *Arthrobacter woluwensis*, respectively. The sequences were submitted to NCBI GenBank with accession no. MW077247 and MW077246 (S. Fig. 3).

### In vitro IAA quantification of isolates ALT29 and ALT43

IAA production in the culture filtrate of isolates ALT29 and ALT43 spiked with different concentrations of NaCl was quantified using GC/MS. ALT29 and ALT43 both produced substantial amounts of IAA, but the highest IAA content was produced by ALT29 (Fig. 1).

### Bacterial isolates ALT29 and ALT43 regulate soybean growth under salinity stress

Salinity stress inhibited the growth attributes and reduced the chlorophyll content of soybean plants. However, the negative effects of salinity were attenuated in soybean plants inoculated with ALT29 and ALT43 (Fig. 2). Compared with the control plants, those exposed to salinity stress exhibited decreases in shoot length (15%), root length (29%), shoot fresh and dry weight (49 and 35%), and root fresh and dry weight (22 and 34%). Inoculation with isolates ALT29 and ALT43 mitigated salinity stress, increasing the shoot length (13%), root length (21%), shoot fresh and dry weight (44 and 35%), and root fresh and dry weight (9%) compared with NaCl-stressed plants (Fig. 2; Table 1). Additionally, under normal condition, increases in chlorophyll content (21%), *Chl a* (10%), *Chl b* (8%), and carotenoid (8%) content were observed in ALT29 and ALT43-inoculated soybean plants compared with control plants (Fig. 3). However, when plants were subjected to NaCl stress (80 mM, 160 mM, and 240 mM), a decrease in chlorophyll content (12–42%), *Chl a* (18–80%), *Chl b* (38–89%), and carotenoid (19–79%) content were observed (Fig. 3). Inoculation with halotolerant ALT29 and ALT43 mitigated the NaCl stress and increased the chlorophyll content (16–24%), *Chl a* (8–43%), *Chl b* (13–46%), and carotenoid (14–39%) content were observed in soybean inoculated plant compared with only NaCl stressed plants (80 mM, 160 mM, and 240 mM) (Fig. 3).

**Table 1:** Growth promoting effect of Isolate ALT29 and ALT43 in soybean under various NaCl stress. The values with ± show standard deviation (SD) are means of three technical replicates. Value in columns followed by different letters are significantly different as evaluated by DMRT analysis

	SL(cm)	RL(cm)	SFW(g/3P)	RFW(g/3P)	SDW(g/3P)	RDW(g/3P)
Cont.	20.26 ± 1.4	24.16 ± 5.34	16.75 ± 0.75	6.50 ± 0.55	1.86 ± 0.45	0.51 ± 0.08
ALT29	23 ± 2.0	25.46 ± 4.88	17.22 ± 1.07	8.54 ± 1.36	2.53 ± 0.45	0.67 ± 0.03
ALT43	22.06 ± 1.0	27.83 ± 5.10	17.07 ± 1.00	7.79 ± 0.71	2.42 ± 0.34	0.63 ± 0.04
80 mM NaCl	16.16 ± 1.04	18.33 ± 0.76	12.50 ± 0.50	5.03 ± 0.50	1.20 ± 0.2	0.38 ± 0.01
80 mM + ALT29	19.96 ± 0.45	20.33 ± 1.10	14.74 ± 0.75	6.87 ± 0.11	2.02 ± 0.10	0.42 ± 0.05
80 mM + ALT43	18.16 ± 1.04	22.23 ± 1.07	14.24 ± 0.96	6.11 ± 0.25	2.06 ± 0.12	0.39 ± 0.11
160 mM NaCl	11 ± 1.00	16.83 ± 0.76	8.52 ± 0.50	3.69 ± 0.20	1.01 ± 0.10	0.42 ± 0.02
160 mM + ALT29	14.1 ± 0.75	19.5 ± 1.50	9.90 ± 0.50	4.99 ± 0.50	1.5 ± 0.11	0.38 ± 0.03
160 mM + ALT43	13 ± 1.01	20.16 ± 0.76	10.28 ± 0.75	5.96 ± 0.54	1.43 ± 0.07	0.52 ± 0.03
240 mM NaCl	8.36 ± 0.77	17.5 ± 0.51	4.22 ± 0.40	2.66 ± 0.32	0.92 ± 0.06	0.34 ± 0.03
240 mM + ALT29	10.5 ± 0.55	20.5 ± 1.00	7.83 ± 0.80	3.01 ± 0.50	1.163 ± 0.14	0.42 ± 0.02
240 mM + ALT43	11.2 ± 0.43	17.73 ± 1.02	8.56 ± 0.51	4.41 ± 0.35	1.14 ± 0.05	0.35 ± 0.01

*SL* Shoot length, *RL* Root length, *SFW* Shoot Fresh weight, *RFW* Root Fresh weight, *SDW* Shoot Dry weight, *RDW* Root Dry weight

### Regulation of endogenous phytohormones under salinity stress

**Fig. 4:**
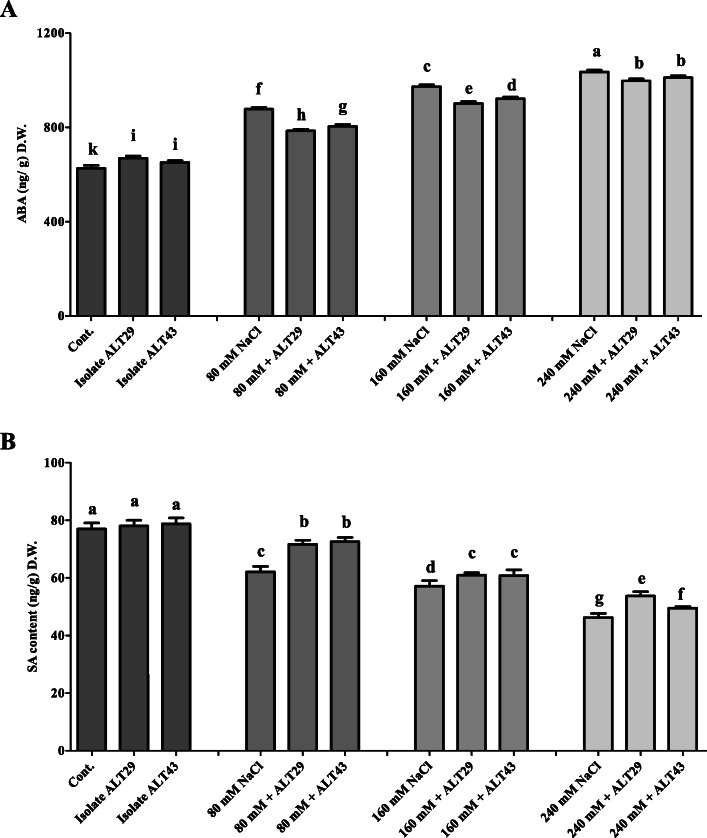
Endogenous abscisic acid (ABA), and salicylic acid (SA) quantification in soybean plants inoculated with ALT29 and ALT 43. **a** ABA content and **b** SA content under normal conditions and NaCl stress. Data points are the mean of three technical replicates. Error bars represent standard error. Bars with different letters are significantly different from each other, as evaluated by DMRT

A significant increase in ABA content (1.57-fold) was observed in soybean plants under salinity stress compared to the control (Fig. 4a). However, among plants exposed to NaCl stress (80 mM, 160 mM, and 240 mM), a decrease in ABA content (0.77-fold) was observed in those inoculated with isolates ALT29 and ALT43 (Fig. 4a). Moreover, SA results were in contrast to ABA results; a 2% increase in endogenous SA content was observed in plants inoculated with ALT29 and ALT43 under normal conditions. Under NaCl stress, a 6–16% increase was observed in soybean plants inoculated with ALT29 and ALT43 (Fig. 4b).

### Antioxidant quantification in soybean plants under salinity stress

**Fig. 5:**
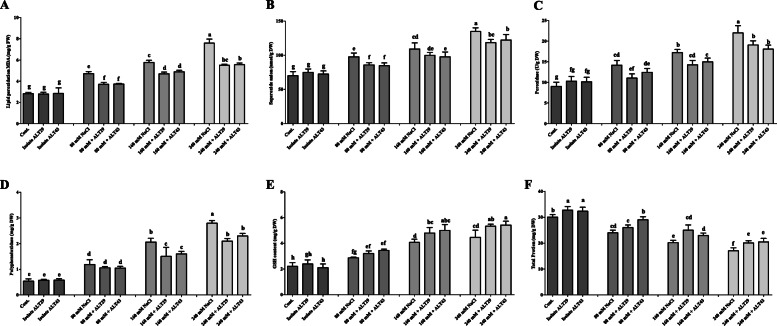
Effects of bacterial isolates ALT29 and ALT 43 on antioxidant content. **a** Lipid peroxidation (MDA); **b** Superoxide anions (SOA); **c** Superoxide dismutase (SOD), **d** Polyphenol oxidase (PPO); **e** Reduced glutathione (GSH); **f** Total protein (TP) content in soybean plants under normal conditions and NaCl stress. Data points are the mean of three technical replications and error bars represent standard error. Bars with different letters are significantly different from each other, as evaluated by DMRT analysis

Changes in antioxidant content were investigated in soybean plants subjected to NaCl stress with and without inoculation with isolates ALT29 and ALT43. Malondialdehyde (MDA) content was evaluated to assess the extent of lipid peroxidation (LPO). Higher levels of MDA (2.3–6.3-fold) were observed in soybeans treated with NaCl stress (80 mM, 160 mM, and 240 mM) compared with ALT29 and ALT43 inoculation (0.8–5-fold) (Fig. 5a). Similarly, superoxide anion (SOA) contents varied in response to the NaCl treatments (Fig. 5b). However, the production of SOA was significantly inhibited in ALT29 and ALT43-inoculated soybean plants (21–68%) compared with NaCl-stressed plants (38–91%). Similar trends were observed in peroxidase dismutase (POD) content and polyphenol oxidase (PPO) content, which were lower in salinity stress soybean plants inoculated with isolates ALT29 and ALT43 (Fig. 5c & d). To further elucidate the ability of ALT29 and ALT43 to mitigate salinity stress, the GSH content in soybean plants was significantly higher (56–179%) in ALT29 and ALT43-inoculated plants compared with un-inoculated plants (37–136%) under NaCl stress (Fig. 5e). In addition, the total protein content significantly decreased (20–43%) under salinity stress compared with control plants. However, inoculation with halotolerant ALT29 and ALT43 increased the protein content in NaCl-stressed plants by 10–20% (Fig. 5f).

### geRole of bacterial isolates in ion uptake during salinity stress

**Fig. 6:**
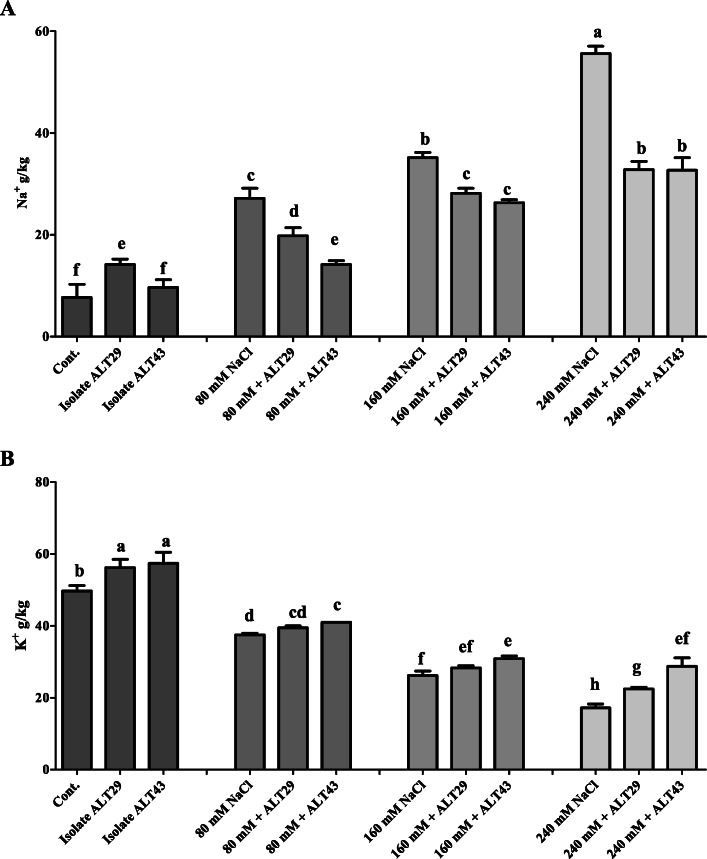
Effects of bacterial isolates ALT29 and ALT43 on sodium (Na^+^) and potassium (K^+^) content. **a**: sodium content (Na^+^); and **b**: Potassium (K^+^) content in soybean plant under normal and NaCl stress. Data points are the mean of three technical replications and error bars represent standard error. Bars with different letters are significantly different from each other, as evaluated by DMRT analysis

Inductively coupled mass spectrometry (ICP) analysis of Na^+^ and K^+^ content was conducted. Soybean plants treated with NaCl (80 mM, 160 mM, and 240 mM) exhibited higher Na^+^ content (4–8-fold) (Fig. 6a). However, ALT29 and ALT43-inoculated plants showed significant decreases in Na^+^ content (2–4.5-fold) (Fig. 6a). Compared with Na^+^, the K^+^ content decreased significantly under salinity stress (24.5–65.39%) compared with control plants (Fig. 6b). However, K^+^ uptake increased (9.34–67.03%) in salinity-stressed soybean plants inoculated with ALT29 and ALT43 compared with un-inoculated plants (Fig. 6b).

### Gene expression under salinity stress and bacteria inoculation

**Fig. 7:**
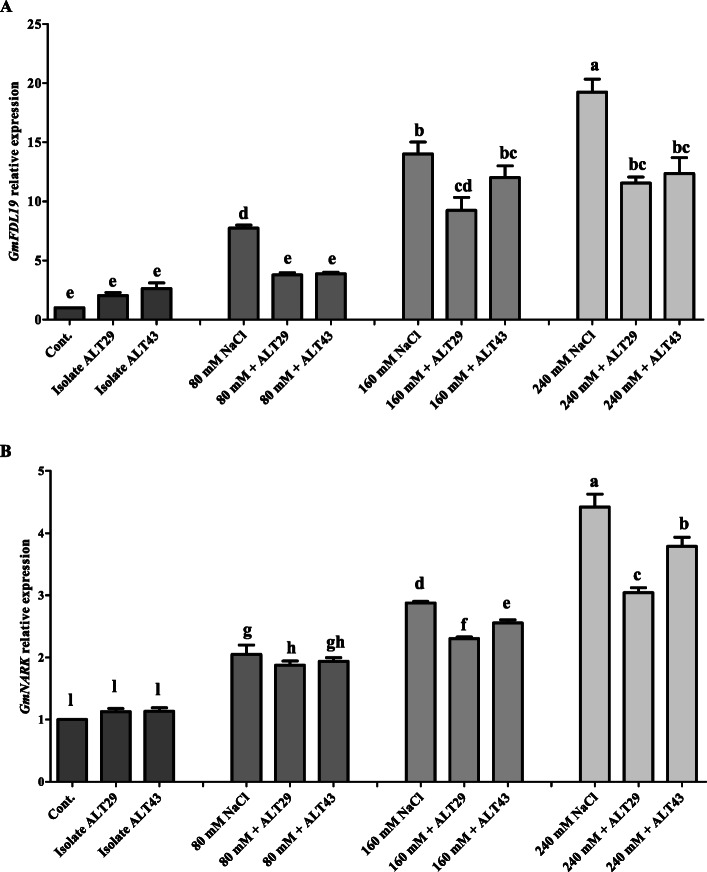
Relative expression of *GmFLD19* and *GmNARK* genes in soybean plants with and without inoculation of ALT29 and ALT 43 under NaCl stress. The values are means of three technical replicates calculated relative to those of actin gene expression. Error bars represent standard error. Bars with different letters are significantly different from each other, as evaluated by DMRT analysis

*GmFLD19* was more highly expressed (7–18-fold) in soybean plants exposed to NaCl stress (Fig. 7a). However, ALT29 and ALT43 inoculation improved soybean adaptability against NaCl stress, resulting in a significant decrease in *GmFLD19* expression (3–12-fold) in soybean plants exposed to NaCl stress (80 mM–240 mM) (Fig. 7a). Similarly, a significant increase in the expression of *GmNARK* was observed in soybean plants exposed to NaCl stress (2–4.6-fold) (Fig. 7b). However, ALT29 and ALT43 inoculation enhanced soybean resistance to NaCl stress and reduced the expression of *GmNARK* (1.8–3.7-fold) in soybean plants exposed to NaCl stress (Fig. 7b).

## Discussion

Soil salinity is a serious agricultural problem around the world [23–25]. In plants, salinity stress can cause oxidative damage, ion toxicity, and nutritional imbalances that reduce photosynthetic rates, inhibit plant growth, and decrease crop yield and quality [17, 30]. Plants under salinity stress undergo several morphological and physiological changes that impede growth and development [30, 32]. However, beneficial plant growth-promoting rhizobacteria have been demonstrated to play a significant role in alleviating salinity stress in plants, improving crop growth and yield in saline environments [17, 30, 32]. Alleviation of salinity stress by PGPR has been reported in cotton, rice, wheat, tomato and soybean [23, 29–36].

In this study, soybean plants exposed to NaCl stress exhibited reduced growth, root and shoot length, and fresh and dry biomass. However, inoculation with bacterial isolates ALT29 and ALT43 mitigated the effects of salinity stress, improving plant-growth attributes in inoculated plants compared to un-inoculated plants (Fig. 2; Table 1). Similarly, chlorophyll content plays a vital role in photosynthesis, however salinity stress affect chlorophyll content depend on level of salinity [37, 38]. A decrease in chlorophyll content was observed in plants under salt stress. However, NaCl-induced decreases in chlorophyll and carotenoid content were attenuated in soybean plants inoculated with ALT29 and ALT43 (Fig. 3). These results are consistent with previous findings that inoculation with halotolerant PGPR increases chlorophyll content in plants under salinity stress, including *Ocimum basilicum* [39], *Solanum lycopersicum* [33], *Glycine max* [34], and *Oryza sativa* [40]. Furthermore Yoo et al. [33] Siddikee et al. [41], Fan et al. [42], and Khan et al. [18] reported *Bacillus aryabhattai* and *Arthrobacter woluwensis* against salinity stress in tomato, canola and soybean plants, and reported that plants inoculated with bacteria isolate significantly enhance plant growth, chlorophyll content under NaCl stress.

The beneficial effects of the selected halotolerant bacterial isolates (ALT29 and ALT43) on soybean growth may be attributed to the salinity tolerance and plant growth-promoting traits (EPS formation, organic acid, and IAA production) exhibited by these isolates (S. Fig. 1 and Fig. 1). Vurukonda [27] and Selvakumar [28] reported that EPS-producing bacteria contribute to the maintenance of salinity tolerance in plants, providing protection against salinity stress [43, 44]. Similarly, organic acids benefit plants by providing an important source of carbon and energy as well as regulating abiotic stress tolerance [45]. The bacteria used in the current study produce several organic acids (Fig. 1b), which have been shown to enhance plant growth. Furthermore, IAA is an essential phytohormone, and numerous studies have demonstrated that IAA-producing halotolerant bacteria improve plant salinity tolerance. In this study, isolates ALT29 and ALT43 produced IAA in LB media with NaCl (Fig. 1a). Therefore, the growth promotion of salt-stressed soybean plants inoculated with ALT29 and ALT43 is likely mediated by these PGP traits.

Salinity stress leads to the accumulation of Na and Cl, which affects the presence and distribution of other vital elements in plants, reducing physiological activity and plant growth [2, 3]. Increased concentrations of Na ions produce osmotic stress, impacting intercellular K influx [6]. K is an essential element for plant growth [46]; the results of this study showed decreases in K and increases in Na in soybean plants under salinity stress (Fig. 6). However, inoculation with ALT29 and ALT43 improved K uptake and reduced Na uptake in NaCl-treated plants (Fig. 6).

In addition, phytohormones are essential signal molecules in plants that are regulated under stress conditions [47, 48]. In particular, salinity stress has been shown to increase the biosynthesis of the phytohormone abscisic acid [49, 50]. Previous studies show that high endogenous ABA content inhibits the growth of *Brassica napus*, *Zea mays*, and *Phaseolus vulgaris* [47, 48, 51]. Inoculation with halotolerant PGPR modulates abiotic stress, including salinity stress, and reduces ABA biosynthesis [35, 52–54]. In the current study, we observed a significant increase in endogenous ABA content in soybean plants under NaCl stress; however, this effect was mitigated by ALT29 and ALT43 inoculation (Fig. 4a). Similarly, salt-stressed *Gossypium hirsutum* and *Triticum aestivum* inoculated with salt-tolerant *Pseudomonas putida*, *Arthrobacter protophormiae*, and *Bacillus subtilis* exhibited reduced ABA accumulation and enhanced plant growth [32, 36].

Salicylic acid is a defense-signaling phytohormone that acts as an ROS scavenger, protecting plants from oxidative stress [55–57]. Hamayun et al. [3] and Wang et al. [58] reported a decrease in endogenous SA in *Glycine max* and *Iris hexagona* under salinity stress. Our results were consistent with these findings in that NaCl reduced SA content in soybean; however, higher SA contents were observed in soybean plants inoculated with isolates ALT29 and ALT43 (Fig. 4b). Our current finding confirms the previous reports of Kang et al. [59] and Khan et al. [60, 61] who reported that bacterial inoculation enhances SA content in plants under abiotic stress.

Accumulation of Na in plant tissues increases the formation of ROS (superoxide anion, singlet oxygen), disturbs the normal metabolism, and produces cellular toxicity and protein degradation [62]. To mitigate the effects of salinity stress and increased ROS generation, plants activate antioxidant defense systems, such as glutathione reductase (GR), LPO, and other antioxidant enzymes (PPO, POD, TPP) that remove free radicals and protect against cellular stress. Soybean plants inoculated with ALT29 and ALT43 exhibited increases in GSH and TPP content and decreases in LPO, SOA, and PPO content under salinity stress (Fig. 5). Similar results were also observed in maize, rice, and basil plants inoculated with halotolerant bacteria, which exhibited significant increases in ROS-scavenging enzyme activity [39, 40, 63, 64].

Furthermore, the identification of genes that confer salt tolerance is essential for genetic engineering of halotolerant crop plants for agricultural production in saline environments. A number of candidate genes have been identified and reported in soybean. *GmFLD19*, a novel group A bZIB gene, was determined to play an important role in abiotic stress tolerance in soybean [15, 65]. *GmNARK* (*Glycine max* nodule autoregulation receptor kinase) also contributes to abiotic stress tolerance and regulates ABA [22]. ABA result of our current investigation showed higher ABA content in NaCl stressed soybean plants might be due to induce in relative expression of *GmNARK* and *GmFLD19* (Fig. 5a). Furthermore, Li et al. [15] also report lower Na content in *GmFLD19* transgenic soybean plants and report that *GmFLD19* might function in regulating absorption of Na by improving salt tolerance in soybean. Our results showed that salinity stress upregulated the expression of *GmFLD19* and *GmNARK* (Fig. 6). However, inoculation with ALT29 and ALT43 stimulated the expression of *GmFLD19* and *GmNARK* in soybean plants exposed to salinity stress (Fig. 6). Previous reports show that overexpression of *GmFLD19* and *GmNARK* in soybean enhances salinity tolerance by reducing Na ion and malondialdehyde content and increasing antioxidant activity and chlorophyll content in transgenic soybean [15, 22]. Overexpression of *GmFLD19* and *GmNARK* in soybean indicate that these genes are exemplary candidates for breeding stress-tolerant cultivars [15, 22].

## Conclusion

The results of the current study show that halotolerant isolates ALT29 and ALT43 possess plant-growth-promoting traits, including salt tolerance, EPS-formation, and organic acid and IAA production. Moreover, ALT29 and ALT43 mitigate the effects of salinity stress and increase growth, biomass, and chlorophyll content in soybean under NaCl stress (80 mM, 160 mM, and 240 mM). The improvement in soybean growth induced by isolates ALT29 and ALT43 can be attributed to the ability of these bacteria to regulate endogenous phytohormones (ABA and SA), antioxidants (GSH, LPO, TPP, PPO, and POD), ion uptake (Na and K), and gene expression (*GmFLD19* and *GmNARK*) under salinity stress. Therefore, we demonstrated that inoculation with ALT29 and ALT43 isolates provides a valuable, ecofriendly, and low-cost biotechnological approach to improve sustainable agricultural production in salt-affected areas.

## Methods

### Isolation, screening, and identification

Rhizospheric bacteria were isolated from Pohang beach, South Korea, as previously reported by Khan et al. [17, 18, 23]. Plants root with adherent soil was transferred into conical flask (99 ml sterilized distilled water and shaken for 3 mints). The sample were diluted through a series of 1-folds dilution, and 0.1 ml of solution was spread on LB agar plates and incubated at 28 °C. Bacterial colonies were collected based on their colony morphology, and preserved in 75% glycerol stock till further analysis.

All isolates were screened for plant growth-promoting traits such as indole-3-acetic acid, siderophore production, phosphate solubilization, EPS production and NaCl tolerant. To assess IAA production, the bacterial isolates were initially evaluated using Salkowski reagent (mix 2 ml 0.5 M FeCl_3_; 49 ml 70% perchloric acid and 49 ml H_2_O) by adding 1 ml supernatant and 1 ml Salkowski reagent and kept in dark for 30 mints. The development of pink color indicated IAA production [27]. For phosphate solubilization Trypticase soy agar medium supplemented with Ca_3_ (PO_4_)_2_ were used for phosphate solubilization, and the plates were incubated at 30 °C for 7 days and observe the formation of transparent halos around each colony [27], while for and EPS formation Congo red assay was used [LB broth (25 g/l), Congo red (0.8 g/l), sucrose (5%), agar (2%)] were assessed according to the methods of and Yuna et al. [66] Furthermore for siderophore production, used chromeazurol “S” agar plates and incubate at 30 °C for the appearance of orange halos in contrast to blue background [27]. Five concentrations of NaCl (purchased from DAEJUNG, Korea) were prepared (0 mM, 70 mM, 140 mM, 210 mM, and 280 mM). A 0.1% culture aliquot was inoculated into 100 ml sterilized LB media and incubated at 30 °C in a shaking incubator before the bacterial density was measured at 600 nm using a spectrophotometer. Based on high salinity tolerance, isolates ALT29 and ALT43 were selected for further evaluation. For identification, genomic DNA and 16S rRNA-specific primers were used and amplified [61, 67]. BLAST NCBI and EzTaxon program were used to determine the homology of different nucleotide sequences of the selected isolates and phylogenetic analysis was conducted using MEGA 6.1 software [68].

### ALT29 and ALT43 IAA and organic acid production

Isolates ALT29 and ALT43 were grown in LB media for 3 days, centrifuged (500×g, 15 min at 4 °C), and analyzed to determine IAA and organic acid content. IAA analysis was conducted following the detailed method of Khan et al. [27]. Bacterial cultural filtrate was filtered through a 0.45 μm cellulose acetate filter, acidified to pH 2.8 and 50 μg/ml [D5]-IAA was added. Furthermore, the culture filtrates were then extracted three time with equal amount of ethyl acetate and completely evaporated through a rotary evaporator. Dried extract was re-dissolved in 5 ml 0.1 M acetic acid and then passed through reverse-phase C18 column. The methanol fractions were prepared by dissolving the residue in 1 ml of methanol and add 1.5 ml diazomethane. The methylated sample were re-dissolved in ethyl acetate and were analyzed by GC-MS SIM to determine IAA content (S. Table 2). The concentration of IAA in the broth was calculated by comparing the peak area of IAA with those of the known standard by GC-MS/SIM. To analyze the organic acid content, the bacterial culture broth was filtered through a 0.22 μl Millipore filter (ADVANTE; DISMIC-25CS) and 10 μl of each sample was injected into a high-performance liquid chromatography (HPLC: Shimadzu Co., Model Prominence) column [waters 600E, included reflective Index detector, RI model RID-10A, Column: PL Hi-Plex H (7.7 × 300 mm]. The isocratic condition for HPLC analysis 0.005 M H2SO4 mobile phase, 0.6 ml/mint flow rate, 65 °C temperature (S. Table 3). Retention times and peak areas of the chromatograms were compared with standards from Sigma-Aldrich, USA [61].

### Growth conditions and treatments

Soybean seeds (Pungsannamulkong variety) were collected from the Soybean Genetic Resource Center (Kyungpook National University Daegu, Republic of Korea). First, seeds were surface-sterilized with 2.5% sodium hypochlorite for 15 min and washed thrice with autoclaved double-distilled water. The sterilized seeds were placed in plastic trays filled with horticulture substrate containing coco peat (45–50%), perlite (35–40%), peat moss (10–15%), and zeolite (6–8%), with NO_3_ (~ 0.205 mg/g), KO (~ 0.1 mg/g), NH^+^ (~ 0.09 mg/g), and PO (~ 0.35 mg/g) [17]. The seedlings were grown in a growth chamber: day/night cycle 14 h at 30 °C/10 h at 25 °C; relative humidity 60–70%; light intensity 250 μmol/m^− 2^ s^− 1^ for 10 days. At the VC stage (unrolled unifoliate leaves), equally-sized seedlings were selected and transferred to plastic pots (10 cm × 9 cm) filled with the same horticulture substrate used in the germination trays.

The experimental design included (a) Control: well-watered (b) Bacterial inoculated ALT29 and ALT43 (c) Treatment 1: 80 mM NaCl stress with or without isolate ALT29 and ALT43 (d) Treatment 2: 160 mM NaCl stress with or without isolate ALT29 and ALT43 and (e) Treatment 3: 240 mM NaCl stress with or without isolate ALT29 and ALT43. To test the plant-protection activity of ALT29 and ALT43 under NaCl stress, 50 ml ALT29 and ALT43 (4.0 × 10^8^ cfu/ml) were inoculated via the drench method and distilled water was used for the control. After 2 weeks, growth attributes (root/shoot length), biomass (fresh/dry weight), and chlorophyll content were measured. A chlorophyll meter (SPAD-502 Minolta, Tokyo, Japan) was used for the chlorophyll measurements. The harvested plants were immediately frozen in liquid nitrogen and transferred to a − 80 °C freeze dryer until further analysis. Following Khan et al. [60], Chlorophyll A and B and total carotenoid were extracted using 80% acetone and measured using a spectrophotometer at absorbances of 663 nm, 465 nm, and 480 nm. Chlorophyll a, b and carotenoid content was calculated using the following formulae:

$$ \mathrm{Chlorophyll}\ \mathrm{a}\ \left(\mathrm{mg}/\mathrm{g}\ \mathrm{FW}\right)=\left[\left\{\left(12.7\ast {\mathrm{A}}_{663}\right)-\left(2.69\ast {\mathrm{A}}_{645}\right)\right\}/1000\ast \mathrm{W}\right]\ast \mathrm{V} $$

$$ \mathrm{Chlorophyll}\ \mathrm{b}\ \left(\mathrm{mg}/\mathrm{g}\ \mathrm{FW}\right)=\left[\left\{\left(22.9\ast {\mathrm{A}}_{645}\right)-\left(4.68\ast {\mathrm{A}}_{663}\right)\right\}/1000\ast \mathrm{W}\right]\ast \mathrm{V} $$

$$ \mathrm{Carotenoids}\ \left(\upmu \mathrm{g}/\mathrm{g}\ \mathrm{FW}\right)={\mathrm{A}}_{480}+\left(0.638\ast {\mathrm{A}}_{663}\right)-\left(0.638\ast {\mathrm{A}}_{645}\right) $$

Where A+ Absorbance at respective wave length; W = fresh weight and V = extraction volume.

### Endogenous abscisic acid and salicylic acid quantification

Endogenous ABA was quantified and extracted according to Qi et al. [69]. For endogenous ABA analysis 3 mg of powdered were treated with 30 ml extraction solution (95% isopropanol and 5% glacial acetic acid) and 10 ng of Me-[2H6]-ABA standard. The suspension was filtered, and the filtrate was concentrated using rotary evaporator. The residue was suspended in 4 ml of 1 N NaOH solution and rinsed trice with 3 ml of methylene chloride in order to eliminate traces of lipophilic material. After decreasing the pH of aqueous phase to 3.5 by adding 6 N HCl, it was extracted through solvent extraction with ethyl acetate trice. The ethyl acetate extract were then evaporated and dry reside was re-suspended in phosphate buffer solution (pH 8), which was passed through polyvinylpolypyrrolidone (PVPP) column. The eluted phosphate buffer solution was once again partitioned 3 time with EtOAc after adjusting pH 3.5 with 6 N HCl. All three aliquots extract were pooled and evaporate through rotary evaporator. The fraction was methylated with diazomethane for detection and ABA was quantified using GC–MS (6890 N network gas chromatograph, Agilent Technologies). Software from ThermoQuest Corp., Manchester, UK, was used to monitor signal ions (m/z 162 and 190 for Me-ABA; m/z 166 and 194 for Me-[2H6]-ABA) (S. Table 4). For the SA analysis, 0.2 g freeze-dried fine powder was mixed with 2 ml of 90 and 100% methanol and centrifuged for 15 min at 10,000×g. The supernatant was evaporated in a vacuum and the sample was re-suspended in 3 ml of 5% trichloroacetic acid. The upper organic layer was then mixed with a solution of isopropanol, ethyl acetate, and cyclopentane (49.5:49.5:1) and vigorously vortexed. The upper layer was transferred to a 4 ml tube and vacuum dried. Prior to HPLC, the dried pellet was mixed with 1 ml of HPLC mobile phase using Shimadzu device outfitted with fluorescence indicator (Shimadzu RF-10AxL) with excitation at 305 nm and emission at. SA was quantified using fluorescence detection (S. Table 5) [70, 71].

### Antioxidant enzyme activities

For protein analysis, frozen plant tissues were ground using an ice-cold pestle and mortar and then added to a solution of 50 mM phosphate-buffered saline, 0.1% polyvinylpyrrolidone (PVP), and 1 mM ethylenediaminetetraacetic acid (EDTA). The homogenate was centrifuged at 10000×g for 10 min at 4 °C. The supernatant was immediately collected and used for protein and antioxidant enzyme quantification. Protein content was measured according to Bradford [72, 73] using BSA as a standard. Superoxide dismutase (SOD) activity was measured according to Khan et al. [60]. LPO, GSH, POD, and PPO activity were determined in accordance with the method described by Chaoui et al. [74] by measuring the absorbance at 290 nm, 470 nm, and 420 nm using a T60 UV-Vis spectrophotometer. POD and PPO activity was determined using the guaiacol method [75], which was performed by adding 0.1 ml supernatant to a reaction mixture containing 1.0 ml of 2% H_2_O_2_, 2.9 ml of 50 mM phosphate buffer (pH 5.5), and 1.0 ml of 50 mM guaiacol. Phosphate buffer was used as a control without enzymes. The absorbance was measured at 470 nm for 3 min, and POD activity was calculated as the unit change per minute.

### RNA extraction, cDNA synthesis, and qRT-PCR analysis

RNA extraction was performed following the protocol of Khan et al. [61]. Briefly total RNA was extracted using 1 ml Trizol® reagent (Invitrogen, USA), from 100 mg crushed leaves of soybean, incubate at room temperature (10mint) and centrifuge (12,000 g; 10 mint; 4 °C). Supernatant were transfer to new tubes (1.5 ml) and add 200 μl chloroform, vortex vigorously (15 s), set on ice (3 mints) and again centrifuged (12,000 g; 15 mint; 4 °C). Transfer upper layer (300-400 μl) to new tube and add 500 μl each of isoproponal and 1.2 M NaCl/0.8 M Na-Citrate, kept for 10 mints at room temperature and centrifuged (12,000 g; 10 mint; 4 °C). The pellet were washed with 1 ml 75% EtOH and centrifuged again (7500 g, 5 mint room temperature), discard supernatant and re-suspend the pellet in 40 μl DEPC water. While A qPCRBIO cDNA Synthesis Kit from PCRBIOSYSTEMS was used for cDNA synthesis. qRT-PCR was performed using a qPCRBIO SYBER Green Kit from PCRBIOSYSTEM, using synthesized (1 μl) of cDNAs as templates and the gene-specific primers. To normalize the level of relative expression of each gene, actin was used for each reaction and the expression level was calculated in control plants relatively with treated plants (S. Table 6). The reaction was performed in a 20 μl volume containing 7 μl ddH2O, 1 μl primer, 10 μl SYBER green and 1 μl cDNA. A total sample volume of 50 μl was subjected to the following condition: initial denaturation at 94 °C for 5 mint, 40 cycle of denaturation at 94 °C for 30 s, annealing at 58 °C for 30 s, extension at 72 °C for 1 mint and final extension at 72 °C for 5 mints [76, 77].

### Determination of Na and K uptake in plant

Na and K content in shoots of bacterial-inoculated and non-inoculated plant samples in the NaCl treatments were investigated according to Khan et al. [61] using inductively coupled plasma mass spectrometry (ICP-MS; Optima 7900DV, Perkin-Elmer, USA).

### Statistical analysis

The results were statistically evaluated by analysis of variance using SAS 9.4 software. All the experiments were repeated three times and data collect from each repetition were pooled together. All data present the mean values with standard error (SE). The mean were analyzed for significant differences among the treatments by using one-way analysis of variance (ANOVA), followed by Duncan’s multiple range tests (DMRT) in SAS (V9.1, Cary, NC, USA).

## Supplementary Information

**Additional file 1.**

## Data Availability

The dataset generated or analyzed and strain used in the current study were submitted to NCBI with the GenBank accession no. MW077246 and MW077247.

## Abbreviations

PGPB: Plant growth-promoting bacteria
IAA: Indole-3-acetic acid
ABA: Abscisic acid
SA: Salicylic acid
APX: Ascorbic acid peroxidase
SOD: Superoxide dismutase
GSH: Glutathione
