# Supplementary material for: Halotolerant bacteria mitigate the effects of salinity stress on soybean growth by regulating secondary metabolites and molecular responses
Source: BMC Plant Biol. 2021 Apr 12;21:176. doi: 10.1186/s12870-021-02937-3 (PMC8040224; doi:10.1186/s12870-021-02937-3)
Supplement: Supplementary file 1 — Additional file 1. [file 12870_2021_2937_MOESM1_ESM.docx]

**Halotolerant Bacteria Reprogram Soybean to Higher Growth Under Salinity Stress by Regulating Secondary Metabolites and Molecular responses**

**Muhammad Aaqil Khan^1^, Atlaw Anbelu Sahile^1^, Rahmatullah Jan^1^, Sajjad Asaf^2^, Muhammad Hamayun^3^, Muhammad Imran^1^, Arjun adhikari^1^, Sang-Mo Kang^1^, Kyung-Min Kim^1^, In-Jung Lee^1*^**

^1^School of Applied Biosciences, Kyungpook National University, Daegu 41566, Republic of Korea

^2^Natural and Medical Plants Research center, University of Nizwa, Nizwa 616, Oman

^3^Department of Botany, Abdul Wali Khan University, Mardan, Pakistan

**S. Fig. 1:** Bacterial isolates assessment for beneficial for plant growth promoting activities. (A) Show LB media plates (B) exopolysaccharide (EPS) activity on Congo red medium, (C) chromeazurol ‘S’ agar plates for siderophore production and (D) Salkowski reagent assay for IAA production.

**
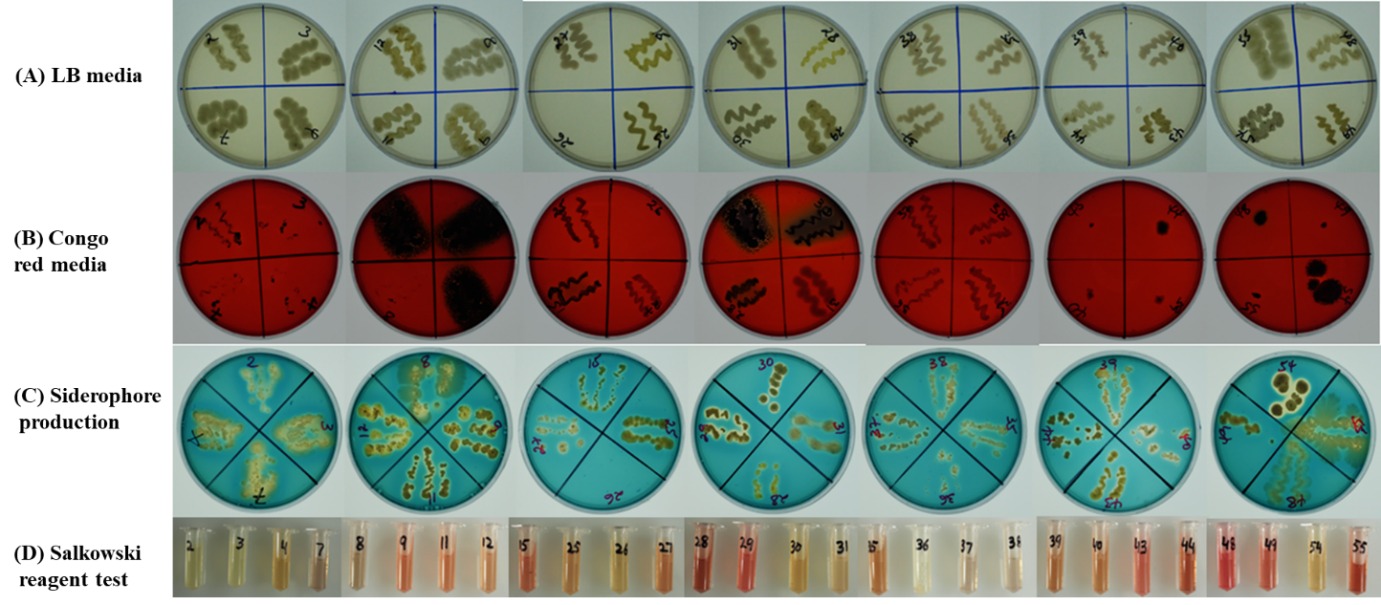
**

**S. Fig. 2:** Growth of multiple plant growth promoting traits producing rhizospheric bacteria. PGPR were grown in LB media at 0 mM, 70 mM, 140 mM, 210 mM and 280 mM NaCl stress for 42 hours and the growth was examined using spectrophotometer at 600 nm. Each data point is the mean of three replication.


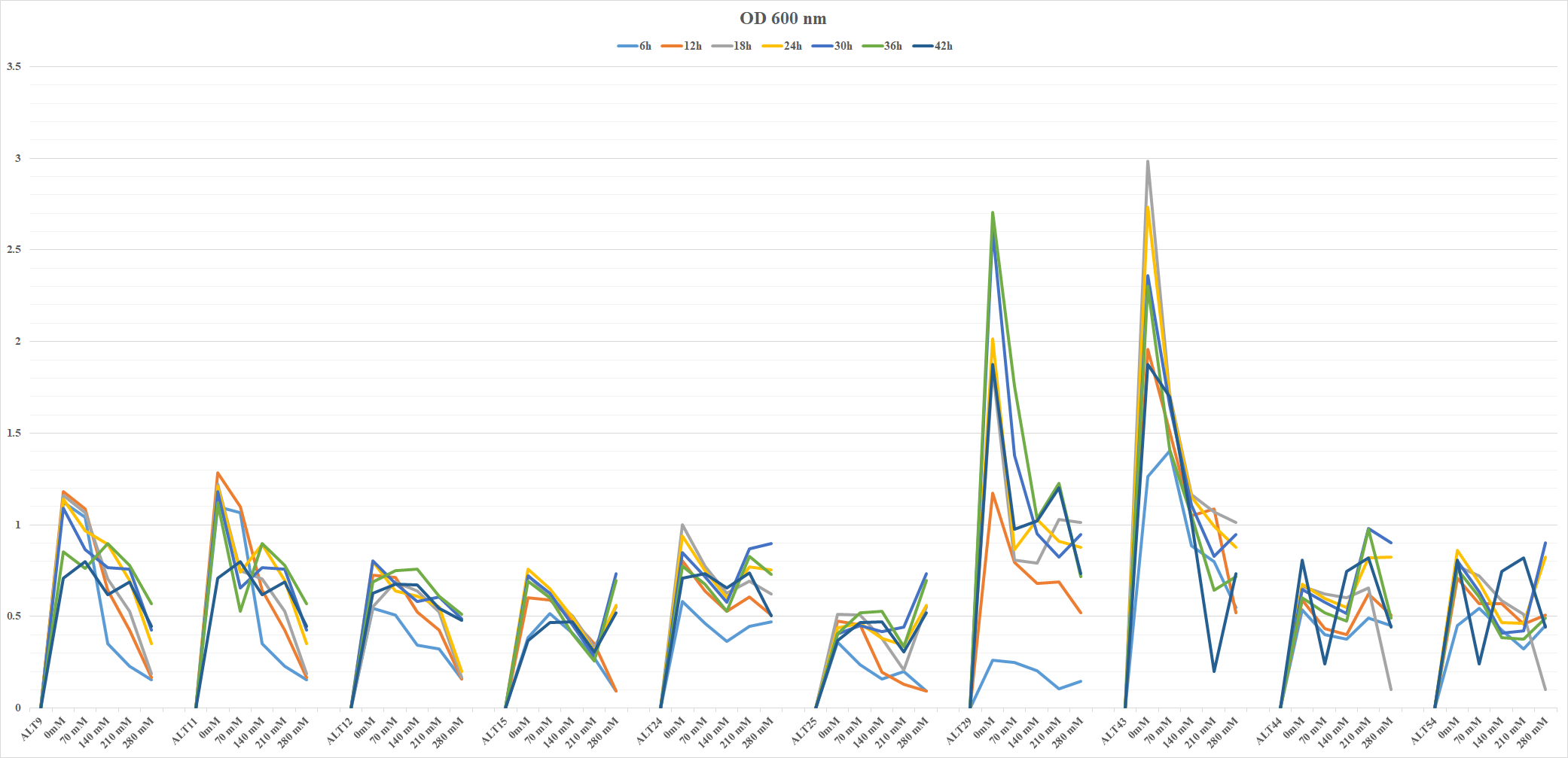


**S. Fig. 3:** Phylogenetic tree of ALT29 and ALT43 was constructed through MEGA 6.0 software by using 16S rRNA sequences by neighbor joining (NJ) and maximum likelihood methods.


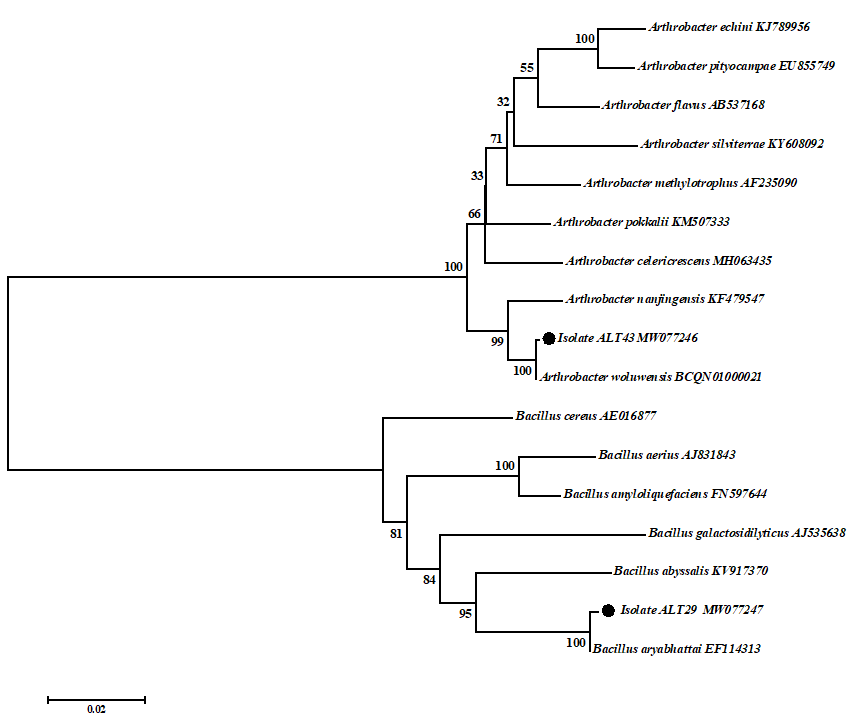


**Supplementary Table 1.** Description of plants species and isolation of rhizospheric bacteria along with their number of yielded isolates having individual or multiple plant growth promoting characteristics.

| **Plants Name** | **No of isolates** | **Isolates having individual Plant Growth Promoting characteristics** | | | **Isolates with multiple plant growth promoting characteristics** |
| --- | --- | --- | --- | --- | --- |
|  |  | **Indol acetic acid production** | **Siderophore** | **EPS production** |  |
| *Artemisia princeps* | 22 | 2 | 4 | 6 | 3 |
| *Chenopodium ficifolium* | 6 | 3 | 4 | 2 | 3 |
| *Oenothera biennis* | 16 | 2 | 1 | 2 | 1 |
| *Echinochloa crus-galli* | 12 | 7 | 2 | 3 | 4 |

**Supplementary Table 2: GC/MS – SIM conditions used for analysis and quantification of the IAA**

| Equipment | Hewlett-Packard 6890, 5973N Mass Selective Detector |
| --- | --- |
| Column | HP-1 capillary column (30m×0.25mm i.d. 0.25 µm film thickness) (J & W Scientific Co., Folsom, CA, USA) |
| Carrier gas | He (60 ml/min.); head pressure of 30 kPa |
| Source temperature | 230°C |
| Oven conditions | IAA: 70°C (2 min.) → 20°C /min. → 280°C (5 min.) |
| Injector temperature | 200°C |
| Ionizing voltage | 70 ev |

**Supplementary Table 3:**

**HPLC conditions used for analysis and quantification of organic acids**

| Equipment | Shumadzu Co., Model Prominence |
| --- | --- |
| Column | PL Hi-Plex H (7.7mm: length, 300mm) |
| Column Temp | 65°C |
| Detector | RI model RID-10A |
| Mobile phase | 0.005M H2SO4 in water |
| Flow rate | 0.6ml/mint |
| Injection volume | 10 μl |

Supplementary Table 4: GC/MS – SIM conditions used for analysis and quantification of the ABA

| Equipment | Hewlett-Packard 6890, 5973N Mass Selective Detector |
| --- | --- |
| Column | HP-1 capillary column (30m×0.25mm i.d. 0.25µm film thickness) (J & W Scientific Co., Folsom, CA, USA) |
| Carrier gas | He (40 ml/min.); head pressure of 30 kPa |
| Source temp. | 250°C |
| Oven conditions | ABA : 60°C (1min.) → 15°C/min. → 200°C →5°C/min. → 250°C →10°C /min → 280°C |
| Injector temp. | 200°C |
| Ionizing voltage | 70 ev |

Supplementary Table 5: HPLC conditions used for analysis and quantification of the SA

| Equipment | Shimadzu LC-10 |
| --- | --- |
| Column | HP hypersil ODS (particle size 5µm, pore size 120Å) |
| Wavelength | Excitation 305nm, Emission 365nm |
| Detector | RF-10Axl (fluorescence detector) |
| Solvent A | 100% MeOH |
| Solvent B | 100% water in 0.5% acetic acid |
| Flow rate | 1.0mL/mint |

**Supplementary Table 6: List of primers used for qPCR analysis**

| ***GmFLD19*** | F | GGTTTGGAGAGATATGCAAC |
| --- | --- | --- |
|  | R | GGCATGTTGTGATGTGTTGT |
| **GmNARK** | F | GAGTTTGGAGACGGGGTGG |
|  | R | CCACTGCCAACACCAACG |
| **GmACT11** | F | ATCTTGACTGAGCGTGGTTATTCC |
|  | R | GCTGGTCCTGGCTGTCTCC |
